# Comparison of the prognostic value of immunoinflammation-based biomarkers in patients with gastric cancer

**DOI:** 10.18632/oncotarget.27653

**Published:** 2020-07-07

**Authors:** Noriyuki Hirahara, Takeshi Matsubara, Yusuke Fujii, Shunsuke Kaji, Yasunari Kawabata, Ryoji Hyakudomi, Tetsu Yamamoto, Takahito Taniura, Yoshitsugu Tajima

**Affiliations:** ^1^ Department of Digestive and General Surgery, Shimane University Faculty of Medicine, Shimane 693-8501, Japan

**Keywords:** gastric cancer, systemic immune-inflammation index, CRP, overall survival

## Abstract

**Background:** Systemic immune-inflammation index (SII)—comprising platelet, neutrophil, and lymphocyte count—is an objective and reliable biomarker for predicting the prognosis in cancer patients because it comprehensively reflects the balance between host inflammatory and immune responses. In this study, we clarified the prognostic impact of immunoinflammation-based indices, i. e. SII, neutrophil/lymphocyte ratio (NLR), and platelet/lymphocyte ratio (PLR), in gastric cancer patients.

**Results:** In multivariate analysis, the American Society of Anesthesiologists physical status (ASA-PS) (hazard ratio [HR]: 3.366, *p* < 0.001), tumor differentiation (HR: 1.705, *p* = 0.020), pathological Tumor, Node, Metastasis (pTNM) stage (HR: 2.160, *p* = 0.008), and carcinoembryonic antigen (CEA) (HR: 1.964, *p* = 0.003) were independent prognostic factors for OS in all patients. Further, multivariate analysis revealed that age (HR: 2.088, *p* = 0.040), ASA-PS (HR: 2.339, *p* = 0.043), tumor differentiation (HR: 1.748, *p* = 0.044), and pTNM stage (HR: 2.114, *p* = 0.024) were independent prognostic factors for OS among patients without inflammation; SII was not a prognostic factor for OS. Meanwhile, body mass index (HR: 5.055, *p* = 0.011), ASA-PS (HR: 3.403, *p* = 0.007), and SII (HR: 4.208, *p* = 0.026) were independent prognostic factors for OS among patients with inflammation.

**Materials and Methods:** We performed a retrospective review of 412 patients who underwent curative laparoscopic gastrectomy. The prognostic value of SII was compared between a low SII group (SII<661.9) and high SII group (SII≥661.9). We analyzed the predictive ability of immunoinflammation-based indices for overall survival (OS) based on a C-reactive protein (CRP) level of 0.5.

**Conclusions:** Compared to NLR and PLR, SII is the most significant prognostic biomarker for OS, especially in gastric cancer patients with inflammation.

## INTRODUCTION

Numerous studies have reported that cancer-related inflammation is an indispensable component of the tumor microenvironment. Cancer causes local or systemic inflammation, ultimately promoting cancer initiation and progression by escaping from the immune system [1–3]. Additionally, systemic immunoinflammation has been generally accepted to affect the cancer microenvironment in a way that favors proliferation, invasion, and migration of cancer cells, while reducing the response to anticancer agents.

Several systemic immunoinflammatory parameters have been evaluated as candidates for predicting long-term survival in various malignancies because systemic immunoinflammation is considered as a consequence, rather than the cause of cancer [4]. Recently, Hu *et al*. demonstrated that the systemic immune-inflammation index (SII) has a strong independent prognostic value in patients with hepatocellular carcinoma treated with surgery [5]. SII comprises 3 peripheral blood parameters, i. e., platelet, neutrophil, and lymphocyte count, which comprehensively reflect the balance of host immune and inflammatory status. In addition, SII is shown to be more objective and reliable for predicting survival in cancer patients than other hematological parameters, including neutrophil/lymphocyte ratio (NLR) and platelet/lymphocyte ratio (PLR), in which both NLR and PLR are based on two inflammatory cells [6–8].

Currently, prognostic prediction in cancer patients mainly depends on the Tumor, Node, Metastasis (TNM) staging system, but the final TNM stage is defined by the histological evaluation of resected specimens after surgery [9]. Preoperative prognostic prediction remains difficult, and the definitive predictor of survival in cancer patients is a subject of ongoing debate. SII is an easily obtained, inexpensive, and non-invasive biomarker that could complement TNM stage in the preoperative prediction of survival in cancer patients. In this study, we clarified the prognostic impact of immunoinflammation-based indices, including SII as well as NLR and PLR, in gastric cancer patients who underwent curative laparoscopic gastrectomy.

## RESULTS

### SII and clinicopathological features in overall patients

The relationships between the SII values and clinicopathological characteristics in the 412 patients enrolled in this study are summarized in Table 1. Based on the SII cutoff value, 307 patients (74.5%) and 105 patients (25.5%) were classified as having a low SII and a high SII, respectively. SII was significantly associated with age (*p* = 0.024), American Society of Anesthesiologists physical status (ASA-PS) classification (*p* < 0.001), BMI (*p* = 0.044), white blood cell count (WBC) (*p* < 0.001), lymphocyte count (*p* < 0.001), neutrophil count (*p* < 0.001), platelet count (*p* < 0.001), tumor size (*p* = 0.002), depth of tumor (*p* < 0.001), lymph node metastasis (*p* = 0.044), pTNM stage (*p* < 0.001), C-reactive protein (CRP) level (*p* < 0.001), NLR (*p* < 0.001), and PLR (*p* < 0.001) (Table 1).

**Table 1:** Relationships between SII and clinicopathological features in overall gastric cancer patients

Characteristics	SII			
	Total patients	< 661.9	≥ 661.9	*p* value
		(*n* = 307)	(*n* = 105)	
Age (years)		70 (36–91)	74 (43–90)	0.024
Gender				0.648
Male	287	212	75	
Female	125	95	30	
ASA				<0.001
1	24	20	4	
2	349	269	80	
3	39	18	21	
BMI		22.5 (14.7–40.4)	21.8 (14.0–32.5)	0.044
WBC		5530 (1830–9280)	6490 (3510–13700)	<0.001
Lymphocyte		1760 (470–3780)	1220 (230–2500)	<0.001
Neutroohil		3169 (1100–6190)	4510 (2650–8494)	<0.001
Platelet		205 (58–460)	251 (119–726)	<0.001
Tumor size (mm)		40 (3–180)	50 (5–170)	0.002
Differentiation				0.305
Well	81	65	16	
Moderate	152	108	44	
Poor	179	134	45	
Depth of tumor				<0.001
T1a-1b	218	180	38	
2	54	42	12	
3	59	37	22	
4a-4b	81	48	33	
Lymph node metastasis				0.044
N0	274	215	59	
N1	50	36	14	
N2	45	27	18	
N3	43	29	14	
Pathological stage				<0.001
1a-1b	247	203	44	
2a-2b	73	49	24	
3a-3c	92	55	37	
CEA antigen (ng/ml)		3.2 (0.7–106)	3.5 (0.8–163.3)	0.144
CRP (mg/l)		0.07 (0.01–6.31)	0.15 (0.01–11.10)	<0.001
NLR		1.826 (0.648–6.893)	4.660 (1.880–16.043)	<0.001
PLR		116.9 (43.8–276.9)	212.0 (99.4–992.7)	<0.001
Postoperative complications				0.087
absent	290	223	67	
present	122	84	38	
Adjuvant chemotherapy				0.133
Yes	114	79	35	
No	298	228	70	

### Cox regression analysis of OS in overall patients

Univariate analyses revealed that worse OS was significantly associated with older age (*p* = 0.006), high BMI (*p* = 0.046), poor ASA-PS (*p* < 0.001), large tumor size (*p* < 0.001), poor differentiation (*p* = 0.004), advanced pTNM stage (*p* < 0.001), high carcinoembryonic antigen (CEA) level (*p* < 0.001), high CRP level (*p* < 0.001), high SII (*p* < 0.001), high NLR (*p* < 0.001), high PLR (*p* < 0.001), postoperative complications (present) (*p* = 0.002), and postoperative adjuvant chemotherapy (yes) (*p* = 0.005). Multivariate analysis revealed that ASA-PS (HR: 3.366, 95.0% CI: 1.917 - 5.911; *p* < 0.001), tumor differentiation (HR: 1.705, 95.0% CI: 1.087–2.674; *p* = 0.020), pTNM stage (HR: 2.160, 95.0% CI: 1.218–3.758; *p* = 0.008), and CEA (HR: 1.964, 95.0% CI: 1.251–3.083; *p* = 0.003) were the independent prognostic factors for OS (Table 2).

**Table 2:** Univariate and multivariate analyses for OS in overall gastric cancer patients

Variables	Patients (*n* = 412)	Category or characteristics	Univariate				Multivariate		
			HR	95%CI	*p* value		HR	95%CI	*p* value
Age	112/300	(<65/≥65)	2.242	1.268–3.962	0.006		1.576	0.870–2.854	0.134
Gender	125/287	(female/male)	1.389	0.865–2.228	0.174				
BMI	372/40	(>18.5/<18.5)	1.785	1.009–3.156	0.046		1.696	0.928–3.100	0.086
ASA	373/39	(<3/≥3)	4.378	2.613–7.336	<0.001		3.366	1.917–5.911	<0.001
Tumor size	244/168	(<5/≥5)	2.275	1.496–3.460	<0.001		1.396	0.836–2.332	0.202
Differentiation	232/180	well & mod/poor	1.85	1.220–2.807	0.004		1.705	1.087–2.674	0.02
pStage	320/92	(1,2/3)	3.647	2.415–5.509	<0.001		2.16	1.218–3.758	0.008
CEA	318/94	(<5.0/?≥5.0)	2.245	1.467–3.436	<0.001		1.964	1.251–3.083	0.003
CRP	350/62	(<0.5/>0.5)	2.452	1.555–3.865	<0.001		1.284	0.750–2.199	0.361
SII	307/105	(<661.9/≥661.9)	2.669	1.755–4.060	<0.001		1.055	0.510–2.182	0.885
NLR	268/144	(<2.529/≥2.529)	2.465	1.630–3.728	<0.001		1.571	0.804–3.069	0.187
PLR	349/63	(<212.1/≥212.1)	2.671	1.680–4.244	<0.001		1.417	0.780–2.573	0.252
Postoperative complications	290/122	(absent/present)	1.982	1.300–3.020	0.002		1.474	0.934–2.325	0.095
Adjuvant	298/114	(No/Yes)	1.814	1.193–2.757	0.005		0.979	0.587–1.634	0.936

### SII and clinicopathological features in inflammation-stratified patients

Based on a CRP level of 0.5 mg/dl, 350 patients (85.0%) were categorized into the non-inflammation group (CRP<0.5) and 62 patients (15.0%) into the inflammation group (CRP³0.5). In patients without inflammation, 274 patients (78.3%) were classified into the low SII group and the remaining 76 patients (21.7%) were classified into the high SII group; the SII was significantly associated with ASA-PS (*p* = 0.044), WBC (*p* < 0.001), lymphocyte count (*p* < 0.001), neutrophil count (*p* < 0.001), platelet count (*p* < 0.001), tumor size (*p* = 0.015), CEA (*p* = 0.041), CRP (*p* = 0.014), NLR (*p* < 0.001), and PLR (*p* < 0.001).

Among the 62 patients with inflammation, 33 patients (53.2%) were classified into the low SII group and the remaining 29 patients (46.8%) were classified into the high SII group. SII was significantly associated with lymphocyte count (*p* = 0.002), neutrophil count (*p* < 0.001), platelet count (*p* < 0.001), depth of tumor (*p* = 0.002), lymph node metastasis (*p* = 0.047), pTNM stage (*p* = 0.001), CRP level (*p* < 0.001), NLR (*p* < 0.001), PLR (*p* < 0.001), and postoperative complications (*p* = 0.024) (Table 3).

**Table 3:** Relationships between SII and clinicopathological features in inflammation-stratified gastric cancer patients

Characteristics	Without inflammation						With inflammation		
	Total patients	SII					SII		
		< 661.9	≥ 661.9			Total patients	< 661.9	≥ 661.9	*p* value
		(*n* = 274)	(*n* = 76)	*p* value			(*n* = 33)	(*n* = 29)	
Age (years)		69.5 (36–91)	73.5 (43–89)	0.081			74 (56–82)	74 (61–90)	0.374
Gender				0.975					0.546
Male	240	188	52			47	24	23	
Female	110	86	24			15	9	6	
ASA				0.044					0.018
1	24	20	4			0	0	0	
2	302	240	62			47	29	18	
3	24	14	10			15	4	11	
BMI		22.5 (14.7–40.4)	21.9 (15.4–32.5)	0.067			22.0 (15.6–28.3)	21.5 (14.0–29.8)	0.703
WBC		5495 (1830–9280)	6480 (4040–10300)	<0.001			6130 (3180–8260)	6730 (3510–13700)	0.066
Lymphocyte		1780 (470–3780)	1220 (230–2270)	<0.001			1660 (800–3180)	1220 (450–2500)	0.002
Neutroohil		3125 (1100–6190)	4530 (2710–7537)	<0.001			3492 (1710–5770)	4440 (2650–8494)	<0.001
Platelet		207 (58–460)	248 (123–665)	<0.001			199 (94–343)	295 (119–726)	<0.001
Tumor size (mm)		38 (4–180)	50 (5–150)	0.015			50 (3–126)	65 (12–170)	0.244
Differentiation				0.501					0.298
Well	73	59	14			8	6	2	
Moderate	123	92	31			29	16	13	
Poor	154	123	31			25	11	14	
Depth of tumor				0.088					0.002
T1a-1b	200	166	34			18	14	4	
2	48	36	12			6	6	0	
3	46	33	13			13	4	9	
4a-4b	56	39	17			25	9	16	
Lymph node metastasis				0.662					0.047
N0	245	196	49			29	19	10	
N1	49	29	11			10	7	3	
N2	30	23	7			15	4	11	
N3	35	26	9			8	3	5	
Pathological stage				0.088					0.001
1a-1b	226	185	41			21	18	3	
2a-2b	59	43	16			14	6	8	
3a-3c	65	46	19			27	9	18	
CEA antigen (ng/ml)		3.1 (0.7–106)	3.6 (0.8–163.3)	0.041			3.7 (1.0–76.3)	2.8 (1.3–29.5)	0.323
NLR		1.784 (0.648–6.894)	3.71 (1.968–16.043)	<0.001			2.196 (0.881–3.667)	3.656 (1.880–6.909)	<0.001
PLR		117.0 (46.7–276.9)	202.1 (99.4–708.7)	<0.001			115.2 (43.8–221.3)	234.0 (119.6–992.7)	<0.001
Postoperative complications				0.955					0.024
absent	257	201	56			33	22	11	
present	93	73	20			29	11	18	
Adjuvant chemotherapy				0.338					0.513
Yes	91	68	23			23	11	12	
No	259	206	53			39	22	17	

### Cox regression analysis of OS in inflammation-stratified patients

In patients without inflammation, univariate analyses identified that a worse OS was significantly associated with older age (*p* = 0.011), poor ASA-PS (*p* = 0.002), large tumor size (*p* < 0.001), poor differentiation (*p* = 0.001), advanced pTNM stage (*p* < 0.001), high CEA level (*p* = 0.001), high SII (*p* = 0.002), high NLR (*p* < 0.001), high PLR (*p* < 0.001), and postoperative adjuvant chemotherapy (yes) (*p* < 0.001). Multivariate analysis revealed that age (HR: 2.088, 95.0% CI: 1.033–4.221; *p* = 0.040), ASA-PS (HR: 2.339, 95.0% CI: 1.026–5.332; *p* = 0.043), tumor differentiation (HR: 1.748, 95.0% CI: 1.014–3.013; *p* = 0.044), and pTNM stage (HR: 2.114, 95.0% CI: 1.103–4.050; *p* = 0.024) were the independent prognostic factors for OS (Table 4). The SII was not confirmed to be an independent prognostic factor for OS.

**Table 4:** Univariate and multivariate analyses for OS in inflammation-stratified gastric cancer patients

Without inflammation											With inflammation							
Variables	Patients (*n* = 350)	Category or characteristics	Univariate				Multivariate				Patients (*n* = 62)	Univariate analysis				Multivariate analysis		
			HR	95%CI	*p* value		HR	95%CI	*p* value			HR	95%CI	*p* value		HR	95%CI	*p* value
Age	100/250	(<65/≥65)	2.395	1.221–4.700	0.011		2.088	1.033–4.221	0.04		12/50	1.511	0.519–4.396	0.449				
Gender	(110/240)	(female/male)	1.266	0.735–2.181	0.395						15/47	1.487	0.559–3.955	0.427				
BMI	318/32	(>18.5/<18.5)	1.63	0.830–3.201	0.156						54/8	3.208	1.050–9.803	0.041		5.055	1.442–17.726	0.011
ASA	326/24	(<3/≥3)	3.3	1.554–7.010	0.002		2.339	1.026–5.332	0.043		47/15	3.711	1.689–8.152	0.001		3.403	1.388–8.342	0.007
Tumor size	219/131	(<5/≥5)	2.515	1.533–4.126	<0.001		1.473	0.813–2.667	0.201		25/37	1.109	0.502–2.452	0.798				
Differentiation	195/55	(well & mod/poor)	2.297	1.384–3.821	0.001		1.748	1.014–3.013	0.044		37/25	1.317	0.602–2.880	0.49				
pStage	285/65	(1,2/3)	3.951	2.421–6.447	<0.001		2.114	1.103–4.050	0.024		35/27	1.989	0.898–4.273	0.091				
CEA	273/77	(<5.0/≥5.0)	2.291	1.388–3.781	0.001		1.703	0.994–2.917	0.053		47/17	2.12	0.932–4.822	0.073				
SII	274/76	(<661.9/≥661.9)	2.229	1.330–3.735	0.002		0.783	0.350–1.751	0.551		33/29	2.776	1.227–6.279	0.014		4.208	1.189–14.887	0.026
NLR	241/109	(<2.529/≥2.529)	2.172	1.327–3.557	0.002		1.755	0.848–3.630	0.13		27/35	2.362	1.016–5.490	0.046		0.602	0.159–2.274	0.454
PLR	306/44	(<212.1/≥212.1)	2.921	1.655–5.157	<0.001		2.068	1.007–4.245	0.058		43/19	1.467	0.649–3.316	0.358				
Postoperative complications	257/93	(absent/present)	1.645	0.977–2.771	0.061						33/29	2.126	0.970–4.661	0.060				
Adjuvant	259/91	(No/Yes)	2.794	1.714–4.554	<0.001		1.355	0.749–2.451	0.315		39/23	0.411	0.165–1.025	0.057				

On univariate analyses for patients with inflammation, OS was found to be significantly associated with high BMI (*p* = 0.041), poor ASA-PS (*p* = 0.001), high SII (*p* = 0.014), and high NLR (*p* = 0.046). Multivariate analysis revealed that BMI (HR: 5.055, 95.0% CI: 1.442– 17.726; *p* = 0.011), ASA-PS (HR: 3.403, 95.0% CI: 1.388 –8.342; *p* = 0.007), and SII (HR: 4.208, 95.0% CI: 1.189 –14.887; *p* = 0.026) were the independent prognostic factors of OS.

### SII and OS across all patients

**Figure 1:**
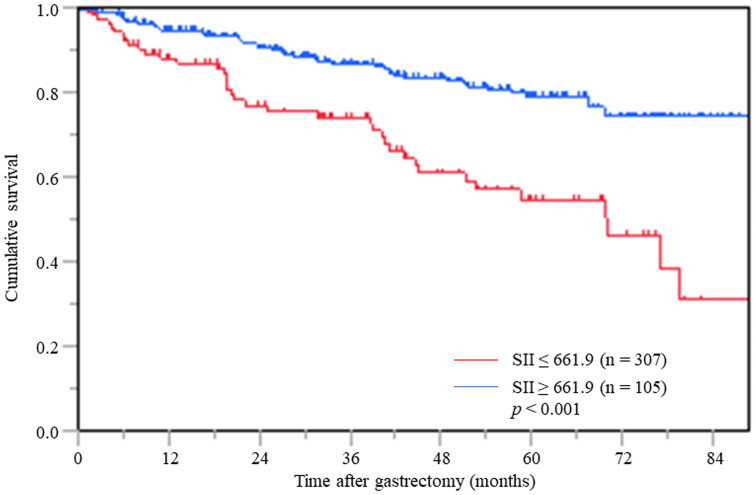
Postoperative OS based on SII in overall gastric cancer patients.

Patients with a low SII had a 3-year OS rate of 86.5% and a 5-year OS rate of 78.9%. In patients with a high SII, the 3-and 5-year OS rates were 74.1% and 54.7%, respectively. The log-rank test demonstrated that patients with a high SII had significantly worse OS than those with a low SII (*p* < 0.001) (Figure 1).

### SII and OS in inflammation-stratified groups

**Figure 2:**
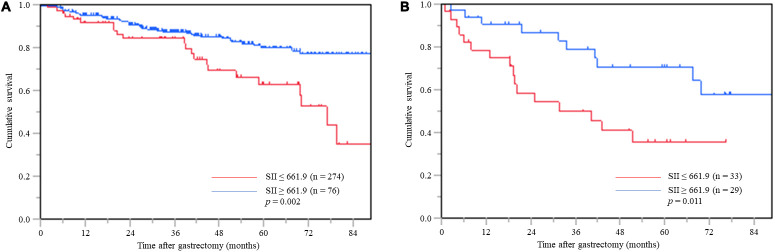
**Postoperative OS based on SII in gastric cancer patients** without inflammation (**A**) and with inflammation (**B**).

In patients without inflammation, the Kaplan-Meier analysis revealed that the 3- and 5-year OS rates in patients with a low SII were 87.5% and 79.9%, respectively, while for patients with a high SII the 3- and 5-year OS rates were 84.3% and 63.0%, respectively. The log-rank test demonstrated that patients with a high SII had significantly worse OS than those with a low SII (*p* = 0.002) (Figure 2A).

In patients with inflammation, the Kaplan-Meier analysis revealed that the 3- and 5-year OS rates in patients with a low SII were 79.0% and 70.7%, respectively, and those in patients with a high SII the 3- and 5-year OS rates were 50.0% and 35.8%, respectively. The log-rank test demonstrated that patients with a high SII had significantly worse OS than those with a low SII (*p* = 0.011) (Figure 2B).

### Predictive ability of SII, NLR, and PLR for OS in inflammation-stratified patients

**Figure 3:**
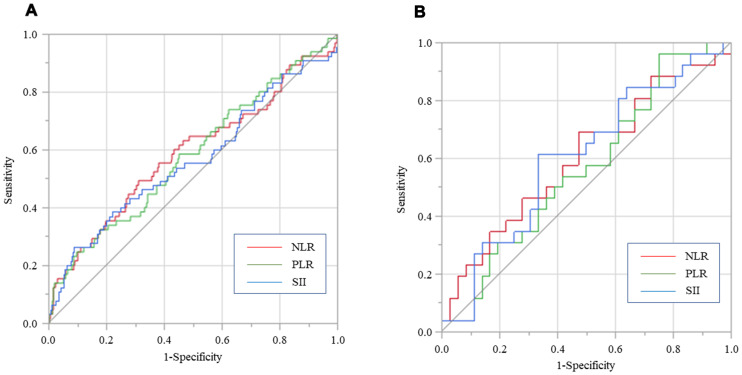
**Predictive abilities of SII, NLR, and PLR for OS examined using ROC curve analysis in gastric cancer patients** without inflammation (**A**) and with inflammation (**B**).

In patients without inflammation, AUCs for SII, NLR, and PLR were 0.565, 0.584 and 0.577, respectively (Figure 3A). Additionally, AUCs for SII, NLR and PLR in patients with inflammation were 0.614, 0.603 and 0.565, respectively. SII consistently had a higher AUC value compare to NLR and PLR in gastric cancer patients with inflammation (Figure 3B).

## DISCUSSION

Previous studies have compared the prognostic impact of inflammation-based parameters, including SII and NLR, in lung, pancreas, ovary, or colon cancer [10–13]. To our knowledge, however, there are no studies on the most suitable parameter for predicting long-term outcomes in gastric cancer. Thus, in this study, we aimed to clarify the clinical and prognostic values of preoperative systemic inflammatory indicators, including SII, NLR, and PLR, in gastric cancer patients who underwent curative laparoscopic gastrectomy.

Neutrophils play crucial roles in the pathogenesis of cancer by enhancing the proliferation, invasion, and metastasis of cancer cells via the release of cytokines and chemokines such as interleukin (IL)-6 and tumor necrosis factor-α (TNF-α). Furthermore, these inflammatory cytokines/chemokines promote angiogenesis and cellular DNA damage, inhibit apoptosis, and protect cancer cells from immune surveillance. As a result, an increasing number of neutrophils can establish a favorable tumor microenvironment and then promote tumor progression [14, 15].

Lymphocytes have an important role in tumor immune surveillance and defense against cancer by inducing cytotoxic cell death and inhibiting tumor cell proliferation and migration. Lymphocytes also block the proliferation and migration of cancer cells by secreting cytokines, such as interferon-γ and TNF-α. Thus, lymphocytes can eliminate cancer cells through cellular and humoral immune mechanisms [16, 17].

Platelets have been proven to induce epithelial-mesenchymal transition via platelet-derived transforming growth factor-β and direct platelet-tumor cell contact. The complex interaction between tumor cells and platelets favors distant metastasis of tumor cells and allows circulating tumor cells to escape from the host’s immune surveillance [18, 19].

Based on the facts mentioned above, previous validations had warranted NLR and PLR, which consist of two types of inflammatory cells, to be well associated with cancer cell behavior and prognosis [10–13]. SII, which comprises 3 peripheral blood parameters, has recently been shown to be a more objective and reliable biomarker for predicting prognosis of cancer patients because it comprehensively reflects both the balance between host inflammatory and immune responses [5, 10].

In our study, a high SII was significantly associated with a larger tumor size, deeper invasion, increased lymph node metastasis, and advanced TNM stage in gastric cancer, indicating a more aggressive tumor phenotype. Therefore, SII could be a beneficial complement to TNM stage in the preoperative prediction of survival in cancer patients.

Virchow has reported that inflammatory reactions and cancer, several studies have found that CRP level is a prognostic factor in a variety of cancer [20]. Systemic inflammatory proteins represented by CRP are synthesized by hepatocytes and induced by proinflammatory cytokines, particularly IL-1, IL-6 and TNF-α [21, 22]. CRP is directly associated with acceleration of angiogenesis, which enhances the progression and metastasis of malignant tumors and contributes to the progression of cancer. CRP is one of the most frequently used serum markers to evaluate prognosis of cancer, but it lacks specificity and could be elevated in a number of systemic influences such as infections, surgery, and connective tissue disease [23–26]. CRP is one of the most frequently used serum markers to assess cancer prognosis, but due to lack of specificity, several studies have been reported, including Glasgow Prognostic Score (Glasgow Prognostic Score (GPS) and CRP/albumin ratio (CAR), which combine CRP and albumin [27–30]. Although the majority of gastric cancer patients have normal CRP, the prognosis prediction by combination of CRP and SII, which comprehensively reflect the balance of host immune and inflammatory status, is important in determining the individualized surveillance and optimized therapeutic strategy to improve prognosis. Inflammatory stratification analysis was performed based on a reference 0.5 mg/dl for CRP, a component of modified GPS reported by Miki et. al [31, 32]. In our inflammation-based cohort, NLR, PLR, and SII were associated with OS in univariate Cox analyses, but they inversely failed to achieve statistical significance in patient without inflammation on multivariate analysis and TNM staging remained independent factor at multivariate. On the other hand, only SII remained as an independent factor on multivariate analysis in patients with inflammation, inversely TNM staging was not associated with prognosis in patients with inflammation. CRP alone is unlikely to be a cancer-specific prognostic predictor, but in patients with inflammation, it was possible to predict cancer-specific prognosis when evaluated in combination with SII. In addition, the comparison of AUC using ROC analysis demonstrated that SII was superior to NLR and PLR for predicting OS after surgery in gastric cancer patients with inflammation. In terms of results, the stratification of prognostic prediction based on SII value is rational especially in patients with non-specific inflammation represented by CRP.

Several limitations of this study should be acknowledged. First, this study was conducted with a small sample size in a single institution, which accounts for the lack of statistical power. Second, there are no universal standard cutoff values for inflammation indices. Most studies determined individual cutoff values by their relevance and significance. As a result, there is a wide range of cutoff values for these indices. Significant cutoff values for inflammatory indices should be verified in properly designed multicentric, independent cohort patients before adopting SII as a predictive biomarker in clinical practice. Third, monitoring of SII during perioperative therapy may provide more important information about the status of systemic inflammatory and immune response as well as therapeutic benefit. In this study, we failed to evaluate postoperative dynamic changes in the SII values. Finally, although SII is a useful and readily available routine blood data, the biological and molecular mechanisms that explain the prognostic predictive nature of SII have not been examined. Furthermore, in overall patients, multivariate analysis showed that SII was an independent prognostic factor for cancer-specific survival, but inflammation stratified analysis did not confirm that SII was an independent prognostic factor for cancer-specific survival (CSS), as only patients without inflammation were significantly associated with CSS (data unshown).

In summary, this study showed that preoperative SII is the most significant prognostic biomarker for OS, especially in patients with gastric cancer with inflammation, when compared to two-factor markers such as NLR and PLR. In the future, properly designed prospective studies should confirm the more significant prognostic value of SII in gastric cancer.

## MATERIALS AND METHODS

### Patients

This retrospective study evaluated 412 consecutive patients who underwent curative laparoscopic gastrectomy for histologically verified gastric adenocarcinoma at our institution between January 2010 and December 2017. Exclusion criteria were as follows: neoadjuvant chemotherapy; active infection within 1 month before surgery; inflammatory, bone marrow, hematological, or autoimmune disease; and a history of other malignancies within the preceding 5 years.

The extent of the gastrectomy and lymph node dissection were determined in accordance with the Japanese Gastric Cancer Treatment Guidelines (version 4) [33]. Similarly, postoperative adjuvant and post recurrence chemotherapy were administered according to the guidelines [33]. Postoperative complications were evaluated according to the Clavien-Dindo (CD) classification and serious complications were defined as grade II or higher [34]. Clinicopathological classification was assessed according to the International Union Against Cancer Tumor, Node, Metastasis (TNM) classification (seventh edition) [9].

The retrospective protocol of this study was approved by the Ethical Review Board of Shimane University, Faculty of Medicine (Shimane, Japan), and the study is registered with the University Hospital Medical Information Network Clinical Trials Registry (UMIN000030472). The requirement for written informed consent was waived because of the retrospective nature of the study.

### Hematological parameter calculation

Preoperative complete blood count (CBC) and blood differential count were derived within 7 days prior to surgery from each patient. The SII was defined as follows: SII = platelet count × neutrophil/lymphocyte count. The NLR and PLR were defined as follows: NLR = neutrophil/lymphocyte count and PLR = platelet/lymphocyte count.

**Figure 4:**
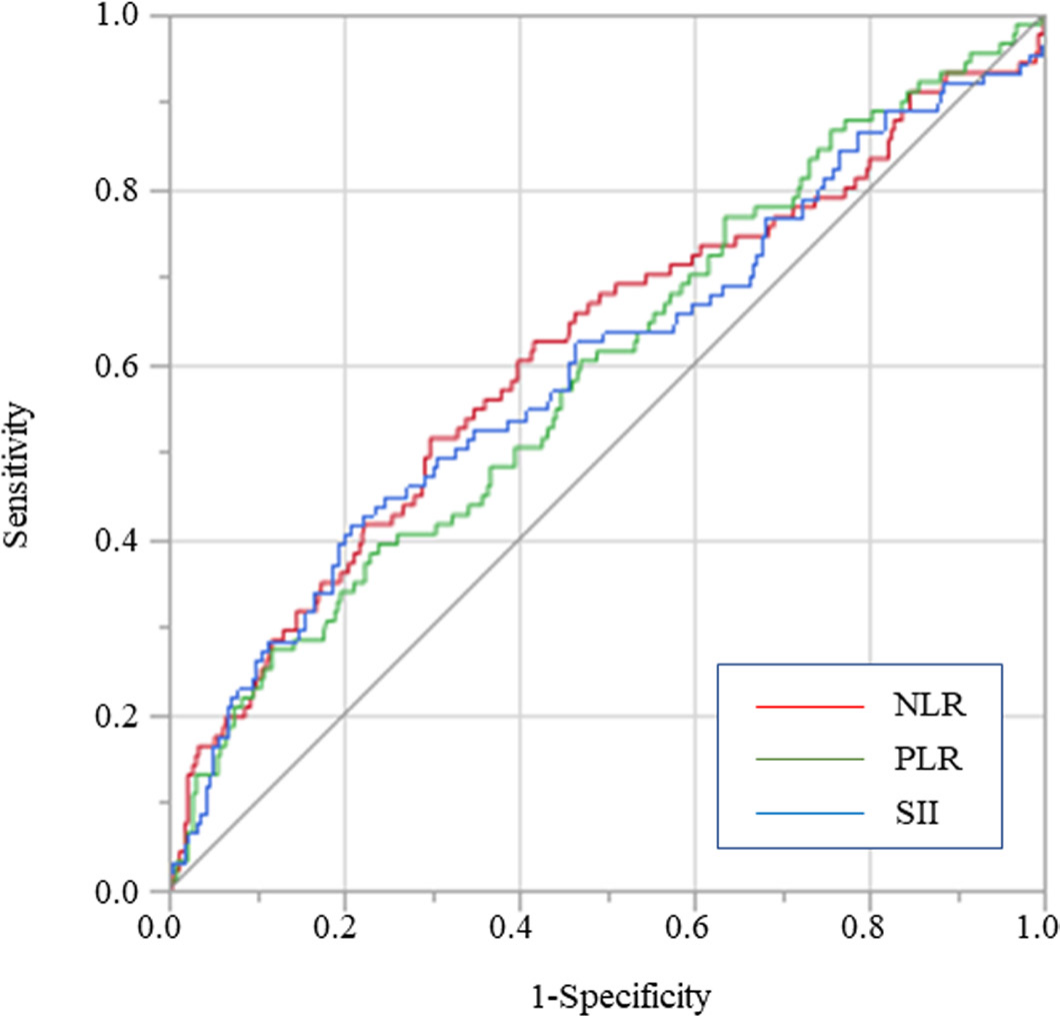
Predictive abilities of SII, NLR, and PLR for OS examined using ROC curve analysis in overall gastric cancer patients.

The receiver operating characteristic (ROC) curve was employed in determining the optimal cutoff value for each inflammation-based index for predicting overall survival (OS) after surgery. The optimal cutoff values were 661.9 for SII, 2.529 for NLR, and 212.1 for PLR, respectively. Consequently, patients were divided into a low or high group according to the individual optimal cutoff values for SII, NLR, and PLR. The area under the curves (AUC) for SII, NLR and PLR were 0.599, 0.613 and 0.593, respectively (Figure 4).

### Follow-up after surgery

Patients were carefully followed up after surgery every 3 months for 2 years, and then every 6 months from years 3 to 5. The routine assessment for recurrence included blood examination, abdominal ultrasonography, chest X-ray imaging, and/or computed tomography. The OS was calculated from the date of surgical resection to the date of death from any cause or the date of last follow-up. The median follow-up duration was 35.9 months (range: 2.7–96.6 months).

### Statistical analysis

The differences between the categorical variables were evaluated by using the Chi-squared test or Fisher’s exact test. The Kaplan-Meier method was used to plot OS after surgery. The differences between survival curves were evaluated via the log-rank test. Cox proportional hazards regression models and Hazard ratios were calculated to test differences between groups. Variables with a *p*-value < 0.05 following univariate analysis were subsequently subject to multivariate logistic regression analysis. All statistical analyses were performed using JMP software (version 15 for Windows; SAS Institute) and a *p*-value < 0.05 was considered as statistically significant.

## Abbreviations

ASA-PS: American Society of Anesthesiologists physical status
AUC: area under the curve
BMI: body mass index
CAR: CRP/albumin ratio (CAR),
CD: Clavien–Dindo
CEA: carcinoembryonic antigen
CRP: C-reactive protein
CSS: cancer-specific survival
GPS: Glasgow Prognostic Score
HR: hazard ratio
IL: interleukin
NLR: neutrophil/lymphocyte ratio
OS: overall survival
PLR: platelet/lymphocyte ratio
RBC: red blood cell
SII: systemic immune-inflammation index
ROC: receiver operating characteristic
TNF-α: tumor necrosis factor-a
TNM: tumor–node–metastasis
WBC: white blood cell count
